# Physiotherapy as a Specific and Purposeful Form of Physical Activity in Children with Idiopathic Body Asymmetry

**DOI:** 10.3390/ijerph192215008

**Published:** 2022-11-15

**Authors:** Jacek Wilczyński, Anita Sowińska, Marta Mierzwa-Molenda

**Affiliations:** Laboratory of Posturology, Collegium Medicum, Jan Kochanowski University, 25-369 Kielce, Poland

**Keywords:** infantile idiopathic asymmetry, body posture of young school children

## Abstract

The aim of the study was to determine the relationship between idiopathic asymmetry in infants and body posture in children at an early school age. The study included 45 girls aged nine. The Diers Formetric III 4D device was used to assess body posture, which allows photogrammetric registration of the back surface using the raster stereography process. For the purposes of the re-search project, the examination was performed via DiCAM using the “Average measurement” mode. Despite physiotherapy, these children had more postural defects later on compared to the control group due to asymmetry. They mainly concerned pelvic skewness, scoliosis angle, deviation from the vertical line and lateral deviation, as well as surface rotation. Positive correlations were observed between direction of asymmetry and pelvic skewness (r = 0.40), and between the location of asymmetry and the location of curvature (r = 0.39). Significant negative correlations were also found between the age of treatment initiation and trunk length (r = −0.42). There was also a negative correlation between the number of physiotherapeutic appointments and deviation from the vertical line, which means that along with an increase in the number of physiotherapeutic visits, the value of deviation from the vertical line decreased (*p* = −0.40). For scoliosis angle, the most important predictor was the direction of asymmetry (*p* = 0.05). For the location of the curvature, the most important predictor was the direction of asymmetry (*p* = 0.04), as well as the number of physiotherapeutic appointments (*p* = 0.04). Additionally, regression analysis allowed us to show that the number of physiotherapeutic visits (*p* = 0.03) was the most important predictor of curvature direction. The applied physiotherapy probably contributed to the occurrence of a smaller number of postural defects in these children at a later age. Physiotherapy as a specific and targeted form of physical activity among infants with idiopathic asymmetry should play a very important role in the prevention of body posture defects.

## 1. Introduction

Physiotherapy, as a specific and targeted form of physical activity for infants with idiopathic asymmetry, should play a very important role in the prevention of body posture defects. Infant asymmetry is a clinical condition that should be handled with great care. It is characterized by a variety of symptoms. It can be seen in posture or motor skills (a decrease or increase in the repertoire of movements), as well as in body composition. Infant asymmetry is further characterized by heterogeneous etiology, location and severity of symptoms. The most common form of infantile asymmetry is idiopathic. It is not based on any disease factors or medical conditions [1,2]. Distinguishing between idiopathic and symptomatic asymmetry is essential for medical and therapeutic interventions. Symptomatic asymmetry is much less frequent [3]. However, it is based on changes that cause functional or structural disturbances. The most frequent asymmetries of this type include: developmental dysplasia of the hip joints, perinatal clavicle fracture, perinatal damage to the shoulder plexus and congenital torticollis. Additionally, it includes damage to the central nervous system, craniosynostosis, congenital, genetically-determined musculoskeletal anomalies, as well as sight- and hearing-related sensory disorders [4,5,6]. In the case of idiopathic asymmetry, its cause is difficult to determine. Environmental factors are often taken into account, such as: the child’s weight, the course of pregnancy, parents’ experience and awareness, infant care methods and the pace of development regarding visual and social functions [7]. Idiopathic asymmetries are rather related to a child’s positioning and side preference. This means that the infant shortens the same side of the trunk and/or the neck during the majority of observation. It is also worth noting that in the first dozen or so weeks of life, an infant may position itself asymmetrically from one side to the other, but this state changes in favor of symmetry. In such a situation, any movements or postural patterns that would be permanent are unlikely to appear. The above-mentioned situation is called physiological asymmetry. The gradual achievement of symmetry is associated with the maturation of the nervous structures and, therefore, with the acquisition of mid-line orientation. Infantile asymmetry is certainly a condition that requires detailed evaluation by a clinician [8,9,10]. The aim of the study was to determine the relationship between idiopathic asymmetry in infants and body posture in children at an early school age. We hypothesized that there was a significant association between idiopathic asymmetry in infants and postural defects in younger school years.

## 2. Materials and Methods

In the study, 45 9-year-old girls were included. The study group comprised 26 individuals who, due to infantile asymmetry, underwent rehabilitation at the Early Intervention Center of the Polish Association for People with Intellectual Disability in Kielce. Inclusion in the study group was based on analysis of medical documentation regarding the physiotherapy process of the child with idiopathic asymmetry and data from a physiotherapeutic interview. The data from the documentation were collected on a medical documentation analysis card containing information obtained during medical examination on admission to the center, physiotherapeutic examination and throughout the course of rehabilitation. However, in the control group, there were 17 girls whose psychomotor development was normal. None of the examined girls entered puberty. The condition for participation was also written consent of the guardian to participate in the project and the acceptance of the child. Parents actively participated in the process of physiotherapy for infants with idiopathic asymmetry. They declared that they strictly followed all the recommendations of the doctor and physiotherapist. The criteria for inclusion in the study were: age 8–9 years, undergoing rehabilitation process in infancy due to idiopathic asymmetry, being born at term, with a high APGAR score, from a properly running, single pregnancy, lack of diagnosed disease syndromes or congenital defects of the CNS and the musculoskeletal system preventing proper psychomotor development, no physical or intellectual disability, no disorders that may be the cause of pathological body posture, i.e.,: genetic syndromes, hormonal disorders, neuromuscular diseases, congenital defects of the locomotor system, and written consent of parents or guardians for testing. The exclusion criteria were: disease syndromes and congenital defects of the CNS or the musculoskeletal system preventing proper psychomotor development, no asymmetry in infancy, improvement, being born prematurely, with a low APGAR score, multiple pregnancy, certificate of physical disability, certificate of intellectual disability, disorders that may be the cause of pathological body posture: genetic syndromes, hormonal disorders, neuromuscular diseases, congenital defects of the locomotor system, the child’s age different than assumed, no written consent for testing.

**Figure 1:**
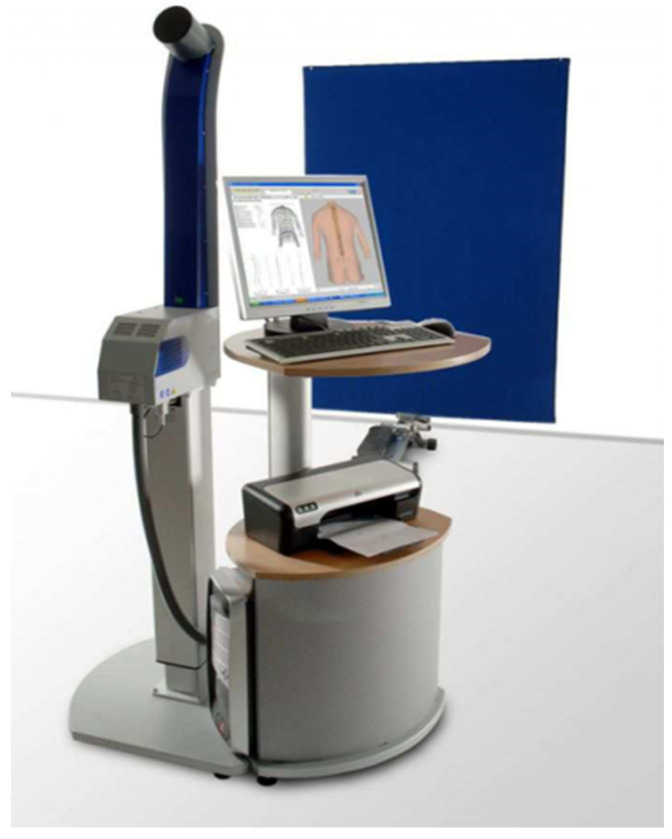
Diers Formetric III 4D.

The study was carried out at the Posturology Laboratory of Collegium Medicum, Jan Kochanowski University in Kielce, in the presence of a parent and in child-friendly conditions. Each participant of the study was informed on the purpose and method of conducting the research and about the possibility of withdrawing from participation at any stage. The trial was approved by the Bioethics Committee No. 25/2018 at the Faculty of Medicine and Health Sciences of Jan Kochanowski University in Kielce. The Diers Formetric III 4D device was used to assess body posture, which allows photogrammetric registration of the back surface using the raster stereography process (Figure 1).

For the purposes of the research project, the examination was performed via DiCAM using the “Average measurement” mode. The following parameters were noted to describe the child’s body posture: pelvic tilt (mm), pelvic tilt (°), lateral deviation (mm) (trunk imbalance), surface rotation (°), deviation from the vertical line (mm), angle of spinal curvature (°), location of curvature, direction of curvature, number of spinal arcs, kyphosis angle (°), lordosis angle (°), trunk length (mm), distance between lumbar dimples (mm), pelvic tilt (°), kyphosis peak (mm), lordosis peak (mm), cervical line (mm) and lumbar line (mm). However, when the curvature of the spine was equal to or greater than 10°, an X-ray was ordered. Scoliosis was diagnosed when the Cobb angle was equal to or greater than 10°.

The distribution of normality was checked using the Shapiro–Wilk test. This test allowed the confirmation of normal distribution. In order to optimize the results, multivariate regression models and one-way ANOVA were applied, as well as correlation analysis. Homogeneity of variance regarding the variables was tested using Levene’s test. In order to determine the predictors for the selected dependent variables (vector R1), the construction of regression models began with the determination of the correlation matrix concerning interdependencies between the analyzed variables. This was carried out according to the following formula:

$$r_{i}=\frac{\sum_{t=1}^{n}\left(y_{t}-y^{-}\right)\left(x_{ti}-x_{i}^{-}\right)}{\sqrt{\sum_{t=1}^{n}{\left(y_{t}-y^{-}\right)}^{2}\sum_{t=1}^{n}{\left(x_{ti}-x_{i}^{-}\right)}^{2}}}$$

Regression models were built in accordance with the definition of the general form of a descriptive (parametric) regression model, for *k* input quantities and *p* parameters. The fit of the model to empirical data was checked with the R2 coefficient of determination. Furthermore, 18 variables were taken into account when building the model. The level of *p* < 0.05 was adopted as the level of statistical significance.

## 3. Results

In infancy, the degree of asymmetry was the same in the left and right directions. The smallest group comprised children who had simultaneous asymmetry of the neck and trunk, but opposite-direction asymmetry was noted in 23.08%. The majority of children (42.31%) exhibited asymmetry located in the neck and trunk at the same time, but in the same direction. Most of the children started physiotherapy at the age of 2 and 4 months (Table 1).

Analysis allowed us to show correct posture in 26.92% of the study group participants. 69.23% demonstrated scoliotic posture while for 3.85%, scoliosis was indicated. In the control group, 36.84% was the observed percentage for correct posture and 63.16% for scoliotic posture (Table 2). Such a large and similar amount of scoliotic posture in the study and control groups is due to the fact that, in general, such posture occurs in a large percentage, even among healthy children.

Among the variables noted for the frontal plane in the study group, the greatest absolute differentiation was shown for scoliosis angle (°) (S = 7.13). The greatest relative differentiation was indicated in the case of pelvic skewness (V = 85.75). In the control group, the greatest absolute differentiation was demonstrated by scoliosis angle (°) (S = 6.50), and the greatest relative differentiation was noted with regard to pelvic skewness (V = 115.41) (Table 3).

Among the variables examined for the sagittal plane in the study group, the greatest absolute differentiation was shown for trunk length (mm) (S = 27.43), while the greatest relative differentiation from the vertical line (mm) was V = 68.92%. On the other hand, in the control group, the greatest absolute differentiation was exhibited concerning kyphosis peak (mm) (S = 231.39), and the greatest relative differentiation was also observed for kyphosis peak (mm) (V = −130.30%) (Table 4).

Positive correlations were observed between direction of asymmetry and pelvic skewness (r = 0.40), and between the location of asymmetry and the location of curvature (r = 0.39). Significant negative correlations were also found between the age of treatment initiation and trunk length (r = −0.42). There was also a negative correlation between the number of physiotherapeutic appointments and deviation from the vertical line, which means that along with an increase in the number of physiotherapeutic visits, the value of deviation from the vertical line decreased (*p* = −0.40) (Table 5).

For scoliosis angle, the most important predictor was the direction of asymmetry variable (*p* = 0.05). For the location of the curvature, the most important predictor was the direction of asymmetry (*p* = 0.04), as well as the number of physiotherapeutic appointments (*p* = 0.04). Additionally, regression analysis allowed us to show that the number of physiotherapeutic visits (*p* = 0.03) was the most important predictor of curvature direction (Table 6).

## 4. Discussion

There is little research in the literature on the relationship between idiopathic asymmetry in infancy and body posture among young school children. Often, idiopathic asymmetries are wrongly perceived as a trivial problem that a child will grow out of with time. However, there is a need to start rehabilitation or at least to inform parents about the principles of proper infant care [8,9,10]. A large number of infants who are referred to a physical therapist due to abnormal postural and movement patterns was the reason to investigate this relationship. Therapeutic experience and research work also indicate that these children have other developmental problems (language, learning difficulties, etc.) [11]. Therefore, it was beneficial to investigate whether this group of children is particularly at risk of developing incorrect body posture in the future. There is not much research on correlations between infantile asymmetry, especially idiopathic asymmetry, and body posture defects. There is also no unanimity as to the causes of this type of asymmetry [12].

According to various sources, this problem affects 8 to 30% of infants within their first months of life [13]. Among the most frequently given reasons for this are functional aspects. The rotation of the head to one side, visible for 3/4 of the observation time, results in the shortening and intensification of muscle tension on the facial side and reduction in tension on the occipital side. Additionally, an asymmetric tonic cervical reflex is triggered. This facilitates the development of childhood scoliosis, increasing tension in the shoulder girdle and neck area, and oblique positioning of the pelvis [14]. It is also worth considering the development of muscle tone, which in an infant developing neurotypically should undergo transformation from proximal mobility and distal stability in the first month of life to proximal stability and distal mobility around its first birthday [15,16]. Based on the sensory information received, the nervous system recruits motor units, determines their mutual coordination and regulates the mutual relationship of muscle groups. That is why oscillatory movements are visible in body posture. These indicate continuous operation of the system based on feedback. Stabilization of individual body segments is also improper in a situation of incorrectly developing postural tension. However, the body posture control system eliminates these deficiencies through the mechanism of passive stabilization [17,18].

The most frequently studied form of infantile asymmetry is plagiocephaly, accompanied by reduced mobility in the neck and slightly delayed gross motor development. Plagiocephaly itself is quite common. According to some studies, it occurs in as many as 40% of healthy-born infants [19,20,21].

The analysis of the results of our own research on body posture in the frontal plane allowed us to show that the values of variables were higher in the groups of children who underwent physical therapy due to asymmetry in infancy. This statement also applies to variables determining the occurrence of scoliotic posture or scoliosis in a child, namely: surface rotation, pelvic skewness and lateral deviation. In the group of children with asymmetry in infancy, there were more cases of scoliotic posture and scoliosis noted than among children who did not demonstrate such asymmetry. Positive correlations were observed between direction of asymmetry and pelvic skewness, and between the location of asymmetry as well as the location of curvature. Significant negative correlations were also found between the age of treatment initiation and trunk length. A negative correlation was further noted between the number of physiotherapeutic appointments and deviation from the vertical line, which means that along with an increase in the number of physiotherapeutic visits, the value of deviation from the vertical line decreased. For scoliosis angle, the most important predictor was the variable regarding direction of asymmetry. For the location of the curvature, the most important predictor was the direction of asymmetry, as well as the number of physiotherapeutic appointments. Additionally, regression analysis allowed us to show that the number of physiotherapeutic visits was the most important predictor of curvature direction.

In the scientific literature on the subject, issues related to body posture in groups of children with diagnosed idiopathic infantile asymmetry have not been adequately described. There is more access to research on the body posture of children with various motor and nervous system problems that begin in infancy. Idiopathic asymmetry is sometimes considered a trivial medical problem that should undergo auto-correction in the course of development [22,23,24]. However, among a large group of children, the beginnings of incorrect posture, or rather movement/postural patterns, were visible from the first weeks of life. Much depends on organization of the health care system in a given country and its ability to identify these infants, referring them to a physiotherapist as early as possible [14,25,26]. A limitation of our research is the lack of continuous analyses regarding the body posture of children aged 2–7 years. The problem of idiopathic asymmetry certainly requires greater vigilance on the part of the clinician and systematic assessment of its impact on body posture.

## 5. Conclusions

Despite the applied physiotherapy intervention, the children under study had more postural defects at an early school age due to asymmetry compared to the control group. These mainly concerned pelvic skewness, scoliosis angle, deviation from the vertical line, lateral deviation and surface rotation. Significant correlations were observed between the features of idiopathic asymmetry and pelvic skewness, surface rotation and location as well as the direction of the asymmetry. For scoliosis angle and location of curvature, the direction of asymmetry was the most important predictor. Infantile asymmetry is a clinical condition that should be handled with great care. The implemented physiotherapy probably contributed to the occurrence of a smaller number of postural defects in these children at a later age. Physiotherapy, as a specific and targeted form of physical activity among infants with idiopathic asymmetry, should play a very important role in the prevention of body posture defects.

## Figures and Tables

**Table 1:** Cross-tabulation of infant idiopathic asymmetry for the study group comprising girls.

**Direction of Asymmetry**				
**Variable**	**Number**	**Cumulative Number**	**Percentage**	**Cumulative Percentage**
Right-sided direction	10	10	38.46	38.46
Left-sided direction	10	20	38.46	76.92
Opposite-direction asymmetry in neck and trunk	6	26	23.08	100.00
**Location of Asymmetry**				
**Variable**	**Number**	**Cumulative Number**	**Percentage**	**Cumulative Percentage**
Located in neck	6	6	23.08	23.08
Located in trunk	9	15	34.62	57.69
Same-direction asymmetry located in neck and trunk	11	26	42.31	100.00
**Age of Beginning Physical Therapy**				
**Variable**	**Number**	**Cumulative Number**	**Percentage**	**Cumulative Percentage**
1 month of life	1	1	3.85	3.85
2 months of life	8	9	30.77	34.62
3 months of life	5	14	19.23	53.85
4 months of life	8	22	30.77	84.62
5 months of life	3	25	11.54	96.15
6 months of life	1	26	3.85	100.00
**Number of Physical Therapy Appointments**				
**Variable**	**Number**	**Cumulative Number**	**Percentage**	**Cumulative Percentage**
2 appointments	2	2	7.69	7.69
3 appointments	4	6	15.39	23.08
4 appointments	3	9	11.54	34.62
5 appointments	4	13	15.38	50.00
7 appointments	3	16	11.54	61.55
8 appointments	4	20	15.38	76.92
9 appointments	2	22	7.69	84.61
10 appointments	1	23	3.85	88.46
11 appointments	2	25	7.69	96.15
12 appointments	1	26	3.85	100.00

**Table 2:** Cross-tabulation for body posture in the frontal plane divided into correct posture, scoliotic pos-ture and scoliosis in the study and control group.

Body Posture of the Studied Girls				
Variable	Study Group		Control Group	
	N	%	N	%
Correct posture	7	26.92	7	36.84
Scoliotic posture	18	69.23	12	63.16
Scoliosis	1	3.85	-	-

**Table 3:** Descriptive statistics regarding the examined variable parameters in the frontal plane among girls from the study and control group.

Variable	Study Group			Control Group		
	X	S	V	X	S	V
Surface rotation (°)	5.00	2.33	46.65	4.00	2.08	52.04
Pelvic skewness (°)	2.54	2.18	85.75	2.21	2.55	115.41
Pelvic skewness (mm)	3.73	2.96	79.35	2.95	3.01	102.08
Pelvic rotation (°)	2.31	1.69	73.30	2.53	1.71	67.75
Location of curvature	2.04	0.82	40.41	1.95	0.78	40.05
Number of arcs	1.00	0.00	0.00	1.0	0.00	0.00
Direction of curvature	1.50	0.51	33.99	1.48	0.51	34.81
Lateral deviation (mm)	4.69	2.77	58.99	3.58	2.41	67.37
Scoliosis angle (°)	13.31	7.13	53.55	10.42	6.50	62.38

**Table 4:** Descriptive statistics regarding the examined variable parameters for the sagittal plane in girls from the study and control group.

Variable	Study Group			Control Group		
	X	S	V	X	S	V
Kyphosis angle (°)	46.15	9.54	20.68	43.45	10.83	24.92
Lordosis angle (°)	41.69	9.98	23.94	39.89	11.83	29.66
Trunk length (mm)	401.54	27.43	6.83	386.00	32.91	8.53
Distance between lumbar dimples (mm)	26.11	11.66	44.63	22.53	3.11	13.83
Pelvic tilt (°)	23.88	4.39	18.40	23.89	4.09	17.14
Kyphosis peak (mm)	−125.19	24.62	−19.67	−177.58	231.39	−130.30
Lordosis peak (mm)	−289.50	23.98	−8.28	−263.00	126.39	−48.06
Pelvic tilt (°)	24.35	7.65	31.41	23.05	5.49	23.82
Deviation from vertical line (mm)	9.00	6.20	68.92	6.95	5.41	77.88

**Table 5:** The results of correlation analysis between body posture and the features of children’s asymmetry in the study group.

Variable	Direction of Asymmetry	Location of Asymmetry	Age of Beginning Physical Therapy	Number of Physical Therapy Appointments
Pelvic tilt (°)	0.40	−0.02	−0.26	0.19
Location of curvature	0.36	0.39	0.21	0.16
Trunk length (mm)	−0.29	−0.01	−0.42	−0.08
Trunk length (mm)	−0.27	−0.02	−0.42	−0.08
Deviation from vertical line (mm)	−0.02	−0.10	0.24	−0.40

**Table 6:** Results of regression analysis for the variables: scoliosis angle as well as the location and direction of curvature in the study group *.

**Angle of Scoliosis**			
**Variable**	**Beta**	**B**	***p***
Constant term	-	13.84	0.07
Direction of asymmetry	−0.01	−0.12	0.05
**Location of Scoliosis**			
**Variable**	**Beta**	**B**	***p***
**Constant term**	-	**1.91**	**0.03**
Direction of asymmetry	0.14	0.15	0.04
Number of physical therapy appointments	0.14	0.04	0.04
**Direction of Scoliosis**			
**Variable**	**Beta**	**B**	***p***
Constant term	-	1.85	0.00
Number of physical therapy appointments	0.21	0.04	0.03

* Y angle of scoliosis = 13.84, −0.12 direction of asymmetry 0.05; Y location of the curvature = 1.91, 0.14 direction of asymmetry 0.04; Y location of the curvature = 1.91, 0.14; number of physiotherapy visits 0.04.

## Data Availability

The data used to support the findings of this study are available from the corresponding author upon request.
